# The IL-33-ST2 axis plays a vital role in endometriosis via promoting epithelial–mesenchymal transition by phosphorylating β-catenin

**DOI:** 10.1186/s12964-024-01683-x

**Published:** 2024-06-10

**Authors:** Jingyao Ruan, Qi Tian, Siting Li, Xiaoyu Zhou, Qianzhi Sun, Yuning Wang, Yinping Xiao, Mingqing Li, Kaikai Chang, Xiaofang Yi

**Affiliations:** 1 Department of Gynecology, Hospital of Obstetrics and Gynecology, Fudan University, 419# Fangxie Road, Shanghai, 200011 China; 2 Department of Pathology, Hospital of Obstetrics and Gynecology, Fudan University, Shanghai, China; 3 Laboratory for Reproductive Immunology, Hospital of Obstetrics and Gynecology, Fudan University, Shanghai, 200011 China; 4 Shanghai Key Laboratory of Female Reproductive Endocrine Related Diseases, Shanghai, China

## Abstract

**Objectives:**

Interleukin 33 (IL-33) is a crucial inflammatory factor that functions as an alarm signal in endometriosis (EMs). Epithelial-mesenchymal transition (EMT), a process related to inflammatory signals, intracellular reactive oxygen species (ROS) production, and lipid peroxidation, have been proposed as potential mechanisms that contribute to the development and progression of EMs. IL-33 is highly upregulated in the ectopic milieu. Moreover, ectopic endometrial cells constitutively express interleukin-33 receptor ST2 (IL-33R). However, the role of IL-33/ST2 in the EMT of EMs remains largely unknown. In this study, we aimed to mechanistically determine the role of IL-33/ST2 in EMs-associated fibrosis.

**Materials and methods:**

We established a non-lethal oxidative stress model to explore the conditions that trigger IL-33 induction. We performed α-smooth muscle actin (α-SMA) protein detection, cell counting kit-8 (CCK-8) assays, and scratch assays to analyze the impact of IL-33 on primary endometrial stromal cells (ESCs) proliferation and invasion. Clinical samples from patients with or without EMs were subjected to immunohistochemical (IHC) and and immunofluorescence(IF) staining to assess the clinical relevance of IL-33 receptor ST2 and EMT-related proteins. Furthermore, we used the ectopic human endometrial epithelial cell line 12Z and normal human epithelial cell line EEC to evaluate the effects of IL-33 on Wnt/β-catenin signaling. The effect of IL-33 on EMT-associated fibrosis was validated in vivo by intraperitoneal injections of IL-33 and antiST2.

**Results:**

We observed that ectopic milieu, characterized by ROS, TGF-β1, and high level of estrogen, triggers the secretion of IL-33 from ectopic ESCs. Ectopic endometrial lesions exhibited higher level of fibrotic characteristics and ST2 expression than that in the normal endometrium. Exogenous recombinant human (rhIL-33) enhanced ESC migration and survival. Similarly, 12Z cells displayed a higher degree of EMT characteristics with elevated expression of CCN4 and Fra-1, downstream target genes of the WNT/β-catenin pathway, than that observed in EECs. Conversely, blocking IL-33 with neutralizing antibodies, knocking down ST2 or β-catenin with siRNA, and β-catenin dephosphorylation abolished its effects on EMT promotion. In vivo validation demonstrated that IL-33 significantly promotes EMs-related fibrosis through the activation of Wnt/β-catenin signaling.

**Conclusion:**

Our data strongly support the vital role of the IL-33/ST2 pathway in EMs-associated fibrosis and emphasize the importance of the EMT in the pathophysiology of fibrosis. Targeting the IL-33/ST2/Wnt/β-catenin axis may hold promise as a feasible therapeutic approach for controlling fibrosis in EMs.

**Supplementary Information:**

The online version contains supplementary material available at 10.1186/s12964-024-01683-x.

## Introduction

Endometriosis (EMs) is a chronic inflammatory, estrogen-dependent condition often accompanied by persistent pelvic pain and infertility [1]. EMs is distinguished by the presence of endometrium-like tissues outside the uterus [2]. Despite its prevalence and impact on women's health [2], its etiology and pathogenesis remain unclear. EMs lesions are clones of specific cells, with variable characteristics as invasive implantation and hormone response. Fibrosis, angiogenesis, and chronic inflammation are the inherent characteristics of endometriotic lesions. Recent research has revealed that endometriotic lesions are located within a highly oxidative stress microenvironment [3]. Reactive oxygen species (ROS) can promote the adhesion and growth of ectopic endometrial cells in the peritoneal cavity, consequently leading to the progression of EMs and disorder-specific symptoms [4, 5]. Recent studies have indicated that oxidative stress is associated with fibrosis in various organs, including the skin, lungs, liver, and kidneys [6–8].

Interleukin-33 (IL-33), a tissue-derived cytokine, is abundantly secreted by various cell types and is recognized as a potent mediator of inflammatory responses and immune regulation [9]. IL-33 can be released in the extracellular space after cell damage (necrotic cell death) or mechanical injury [10, 11], and binds to its receptor ST2. Thus, it was act as a alarmin to alert the immune system of tissue damage upon infection or injury [12]. IL-33 is secreted into the respiratory system in response to oxidative stress [13]. IL-33/ST2 signaling has been implicated in tissue repair, fibrosis, and modulation of immune responses [14, 15]. Furthermore, IL-33 plays a vital role in regulating type 2 innate lymphoid cells [16–20] and promotes the invasion of endometrial stromal cells (ESCs) through the ST2/MAPK/MMP9 pathway [21] in EMs.

Epithelial-mesenchymal transition (EMT) is a process in which epithelial cells acquire mesenchymal features [22, 23]. EMT plays pivotal roles in the pathogenesis of inflammation and fibrosis [24]. Wnt/β-catenin pathways are the major venues triggering EMT [22]. Ectopic endometrial lesions show increased expression of mesenchymal markers and downregulated expression of epithelial markers [25]. The involvement of Wnt/β-catenin in EMs pathogenesis, particularly in the context of IL-33 signaling, presents an intriguing area of investigation.

In this study, we elucidate a novel insight that IL-33/ST2/β- catenin signaling strongly enhances EMT in EMs. Targeting IL-33/ST2 signaling may hold further research avenues for non-hormonal interventions during EMs-associated fibrosis.

## Materials and methods

### Clinical Sample preparation

This study was approved by the Ethics Committee of the Obstetrics and Gynecology Hospital of Fudan University (2020–137). Ethical approval was obtained and all patients enrolled signed an informed consent before being enrolled in the study. The patient consents to the collection of tissue samples and clinical information without prejudice to the pathological diagnosis. Ectopic endometrial lesions and peritoneal fluid samples were obtained from 26 patients with pathologically confirmed EMs. Peritoneal fluid and normal endometrial samples were obtained from 22 patients who were treated laparoscopically and hysteroscopically for tubal infertility or uterine mediastinum. One portion of the biological specimen was used for primary cell extraction and culture, and another portion was fixed in formalin and embedded in paraffin blocks.

### Primary Endometrial Stromal Cell (ESC) culture

The endometrial tissues and ectopic endometrial lesion (from women with endometriomas) were collected under sterile conditions and transported to the laboratory on ice in Dulbecco’s modified Eagle’s medium (DMEM)/F-12 (Hyclone, Logan, UT, USA) supplemented with 1% penicillin, streptomycin and amphotericin B (Sangon Biotech, Shanghai, CN). The primary ESCs were obtained according to previously described methods [26, 27]. In brief, endometrial tissues were cut into small pieces and then digested by stirring in a DMEM/F12 medium with collagenase type IV (0.1%; Sigma-Aldrich; Merck KGaA) at 37℃ for 30–60 min according to the the amount of tissues to be digested. The resulting suspension was filtered in turn through sterile filter (pore diameter size: 40, 100µM) to remove undigested tissue debris. Cells and digestive fluid were centrifuged at 1000rpm for 5 min at 4 °C to remove the supernatant. Cell pellet was further resuspended in red cell lysis buffer for 1 min to remove erythrocytes. After cell centrifugation of 1000rpm for 5 min at 4 °C, cells were resuspended in DMEM/F12 (Hyclone, Logan, UT, USA) containing 10% fetal bovine serum (Gibco, Grand Island, NY, USA) with 1%penicillin, streptomycin and amphotericin B (Sangon Biotech, Shanghai, CN) and seeded in culture flasks that were incubated at 37 °C in 5% CO2 incubator overnight. ESC was attached to the wall and white blood cells were suspended in the medium. On the second day, the cell supernatant was discarded and the new medium was replaced. Finally, the primary ESCs were used for the subsequent experiments.

### Human Endometrial Epithelial Cell Line(EEC)/endometriotic cell line (12Z) culture

The human normal endometrial epithelial cell line (EEC, WHELAB C1225) was sponsored by Shanghai WHELAB BIOSCIENCE Limited and immortalized human endometriotic cell line (12Z) was purchased from Applied Biological Materials Limited and cultured in Minimum Essential Medium (MEM, Corning, NY, USA) supplemented with 10% fetal bovine serum (Gibco, Grand Island, NY, USA) and 1% penicillin/streptomycin/amphotericin B (Sangon Biotech, Shanghai, CN) at 37 °C in a 5% CO2 incubator. Cells were treated with human recombinant IL-33 (10 ng/mL, Peprotech, Waltham, Massachusetts, USA) and ST2 neutralizing antibody (1μg/mL, R&D Systems, Minnesota, USA) for 24 h. PKA and AKT were inhibited using H-89 (5 μM, Sigma-Aldrich; Merck KGaA) and MK-2206 (500 nM, MedChemExpress, New Jersey, USA), respectively.

### Evaluation of IL-33 mRNA and protein level

The primary intrauterine ESCs from patients with or without EMs, as well as the ectopic ESCs from EMs were treated with H_2_O_2_(1µM), TGF-β1(10ng/ml, Peprotech, Waltham, Massachusetts, USA) or estradiol (10^−7^M, Sigma-Aldrich; Merck KGaA) for 24 h, respectively.

### Real-time Polymerase Chain Reaction (real-time PCR)

The primary ESCs in each group and EEC/12Z were collected and used for RNA extraction. RT-qPCR was executed to validate mRNA levels of IL-33. In brief, total RNA was isolated using the EZ-Press RNA Purification Kit (EZBioscience, California, USA), and cDNA was synthesized using PrimeScript™ RT Master Mix (Takara, Japan). Real-time PCR was performed using gene-specific primers and Hieff UNICON® Universal Blue qPCR SYBR Green Master Mix (Yeasen, Shanghai, China). Gene expression levels were normalized to an internal control, and relative expression was calculated using the 2^−ΔΔCt^ method. The primers used are listed in Supplementary Table 2.

### Enzyme-linked immunosorbent assay (ELISA)

Concentrations of IL-33 in cell surpernatant and Peritoneal fluid were determined by commercially ELISA systems using the protocol recommended by the manufacturer (biolegend,CA,USA). Concentrations of CCN4 in cell surpernatant were determined by commercially ELISA systems using the protocol recommended by the manufacturer (RUIXIN BIOTECH, Quanzhou, China). The absorbance was measured at 450 nm with a Synergy H1 Multi-Mode Microplate Reader (BioTek, USA).When running an ELISA, the values of the samples are assigned in relation to the standard curve. Samples were diluted, the concentrations read from the standard curve were multiplied by the dilution factor. Always run ELISA samples in duplicate or triplicate. This will provide enough data for statistical validation of the results. Average the duplicate or triplicate readings for each standard, control, and sample and subtract the average zero standard optical density (O.D.).

### Cell viability assay

Cell viability was assessed using the cell counting kit-8 (CCK-8) assay (Dojindo, Japan). Approximately 100 µL of cell suspension per well was inoculated in a 96-well plate, and the procedure was repeated three times. After treatment, 10 µL CCK-8 solution was added to each well of a 96-well plate, and the cells were incubated for 2 h. Absorbance at 450 nm was measured using a microplate reader. Cell viability (%) = [(A_sample_ – A_blank_)/(A_standard_ – A_blank_)] × 100.

### Quantitative analysis and establishment model of oxidative stress

A lipid peroxidation product, 4-hydroxy-2-nonenal (4-HNE), was used for quantitative analysis of oxidative stress in clinical samples. To assess the generation of ROS, a fluorescent probe such as DCFH-DA (2',7'-dichlorodihydrofluorescein diacetate) (Beyotime Biotechnology, Shanghai, China) was used. ESCs were treated with 0.1–5 µM hydrogen peroxide (H_2_O_2_) for 24 h, washed, and then incubated with DCFH-DA for 20 min at 37 °C and assessed by CCK-8 assay. The fluorescence intensity, indicating ROS levels, was measured using a fluorescence microplate reader (488 nm excitation wavelength and 525 nm emission wavelength) or a fluorescence microscope. Based on the results of CCK8 and DCFH-DA, the treatment concentration of H_2_O_2_ was determined to be 1 μM. A cellular glutathione peroxidase assay kit with NADPH, catalase assay kit, and total superoxide dismutase assay kit with WST-8 (Beyotime Biotechnology, Shanghai, China) were used to detect the related antioxidant enzymes. A lipid peroxidation malondialdehyde (MDA) assay kit (Beyotime Biotechnology, Shanghai, China) was used to detect lipid peroxidation.

### Scratch wound healing assay

The cells were seeded in a culture dish and allowed to form a monolayer. A sterile pipette tip was used to create a uniform scratch (wound) across the cell monolayer. The cells were washed to remove debris, and fresh serum-free medium was added. Images of scratches were captured at various time points. The percentage of wound closure was quantified using image analysis software (Image Pro Plus 6.0, Media Cybernetics, USA).

### Construction of tissue microarrays (TMAs)

TMAs were established in our previous study [28]. All ectopic tissues used for the TMAs construction were diagnosed as EMs by an experienced gynecological pathologist. Additionally, atypical hyperplasia or malignancy of the endometrium was excluded during TMAs construction. In total, 142 formalin-fixed, paraffin-embedded tissues (97 EMs lesions, 34 endometrial samples from EMs, and 11 normal endometrial samples from controls) were included in the TMAs. Multiple 4-μm sections of TMA were used for immunohistochemistry.

### Immunohistochemistry (IHC)

The TMAs were deparaffinized, rehydrated, and subjected to antigen retrieval. Sections were blocked with Tris buffered saline (TBS) containing 1% BSA(Yeasen, Shanghai, China) and 10% goat serum (Sangon Biotech, Shanghai, China) for 60 min. For blocking, the primary antibody was prepared in an antibody dilution buffer. Primary antibodies were applied overnight at 4 °C. The sections were then washed three times with TBS and 0.1% Tween-20 (TBST).The sections were blocked with 3% H_2_O_2_ for 15 min for IHC. After washing, the sections were incubated with HRP-secondary antibodies and developed using a DAB substrate. The slides were counterstained with hematoxylin and examined under a microscope. The average optical density was measured using Image-Pro Plus 6.0.

The following IHC antibodies were used: anti-human ST2 (1:200), anti-human CCN4 (1:200), and anti-human E-cadherin (1:500) (abcam, Cambridge, UK); anti-human vimentin (1:100) (Cell Signaling Technology, Boston, USA); anti-human/mouse Fra-1 (1:200), anti-mouse CCN4 (1:100), and anti-mouse vimentin (1:100) (Affinity Biosciences, China); The primary antibodies used are listed in Supplementary Table 1.

### Immunocytochemistry (ICC)/ Immunofluorescence (IF)

Cell specimen: Cells were fixed with 4% paraformaldehyde for 10min, and permeated with 0.5% triton X-100 at room temperature for 20min. Paraffin-embedded tissue sample: The TMAs were deparaffinized, rehydrated, and subjected to antigen retrieval.

And then, the cells and TMAs were blocked TBS containing 1% BSA(Yeasen, Shanghai, China) and 10% goat serum(Sangon Biotech, Shanghai, China) for 60 min. While blocking, prepare primary antibody in Antibody Dilution Buffer. Primary antibodies were applied overnight at 4 °C. After washing, the cells and TMAs were incubated with secondary antibodies for 2h. The cells and TMAs were counterstained with DAPI and examined under a fluorescence microscope.

The following ICC/IF antibodies were used: anti-human Phospho-B-Catenin (Ser675) (1:200) and anti-human Phospho-B-Catenin (Ser552) (1:200) (Cell Signaling Technology, Boston, USA); anti-human E-cadherin (1:200), anti-human Vimentin (1:1000), and anti-human ST2 (1:50) (Abcam, Cambridge, UK). The primary antibodies used are listed in Supplementary Table 1.

### Western Blotting (WB)

Total protein was extracted from the cells using RIPA Lysis buffer (Beyotime Biotechnology, Shanghai, China) with a protease inhibitor cocktail (TargetMol Chemicals, Shanghai, China), separated by SDS-PAGE (NCM Biotech, Suzhou, China), and transferred to an Immobilon-P Transfer Membrane (Merck Millipore Limited, Germany). The membranes were probed with primary antibodies against the target proteins and then with HRP-conjugated secondary antibodies. The protein bands were visualized using enhanced chemiluminescence (ECL) (NCM Biotech, Suzhou, China) and quantified.

The following WB antibodies were used: anti-human vimentin antibody (1:1000), anti-human N-cadherin antibody(1:1000), anti-human E-cadherin antibody(1:1000), anti-human β-catenin antibody(1:1000), anti-human Fra-1 antibody(1:1000), anti-human phospho-β-catenin (Ser675) antibody(1:1000), anti-human phospho-β-catenin (Ser552) antibody(1:1000), anti-human phospho-Akt (Ser473) antibody, anti-human Akt (pan) (C67E7) antibody(1:1000), and anti-human CREB (48H2) antibody(1:1000), and anti-Akt (pan) (C67E7) antibody(1:1000) (Cell Signaling Technology, Boston, USA); anti-human CREB (phospho S133) antibody (1:5000), anti-alpha smooth muscle Actin antibody (1:10,000) and anti-human GAPDH antibody(1:10,000) (Abcam, Cambridge, UK). All primary antibodies used in WB are listed in Supplementary Table 1.

### Masson's trichrome staining

Masson's trichrome staining was performed to assess tissue fibrosis. Sections were deparaffinized, rehydrated, and stained using a Masson's trichrome staining kit (BASO, China) according to the manufacturer's instructions. Collagen deposition was visualized under a light microscope.

### Small interfering RNA (siRNA) Knockdown of ST2 and β-catenin

siRNAs against human IL1RL1 and CTTNB1 were obtained from Shanghai Generay Biotech (Shanghai, China). The siRNAs were transfected into cells using Lipofectamine 3000 (Thermo Fisher Scientific, Waltham, Massachusetts, USA) according to the manufacturer’s instructions, and the knockdown efficiency was confirmed by WB. The siRNA sequences are listed in Supplementary Table 3.

### Allograft mouse model of EMs

All animal experiment protocols were approved by the Animal Experimental Committee of Fudan University (2022JS). BALB/c mice aged 6 weeks were obtained from the Shanghai JieSiJie Laboratory (China). Before starting the experiments, the animals were acclimatized for 7 days and maintained under controlled temperature and light cycles (24 °C and 12/12 h light/dark cycle) with free access to regular food and water. The cages were changed every week.The following week, the mice were administered two injections of estradiol (0.3μg/mouse) into the muscles of the left hind limb to promote endometrial thickening. The modeling process followed the protocol described in previous literature [19]. Briefly, the uterus of a donor mouse was surgically removed, and cut into fragments smaller than 3 mm with scissor, suspended with 400ul phosphate buffer saline (PBS). Total 400ul of uterine fragment suspension was absorbed with sterile syringe and evenly injected into the peritoneal cavity of 2 recipient mice, 200ul/each mouse. After the injection, abdomen was gently rubbed to make the uterine fragments evenly dispersed. The procedure was repeated for the rest mice. After all the 28 recipient mice were injected with uterine fragments, they were placed in the same feeding box and randomly divided into four groups again to reduce differences and confounding factors. The mice were then treated with intraperitoneal injections of exogenous recombinant mouse IL-33 at 100 ng/mouse (PeproTech, Waltham, Massachusetts, USA) and/or ST2 neutralizing antibody at 10 µg/mouse (R&D Systems, Minnesota, USA) every two days for 28 days following surgery. After 28 days, the lesions were harvested, counted, weighed, and Masson and IHC staining were performed.

## Results

### High expression of IL-33 in ectopic milieu

**Fig. 1:**
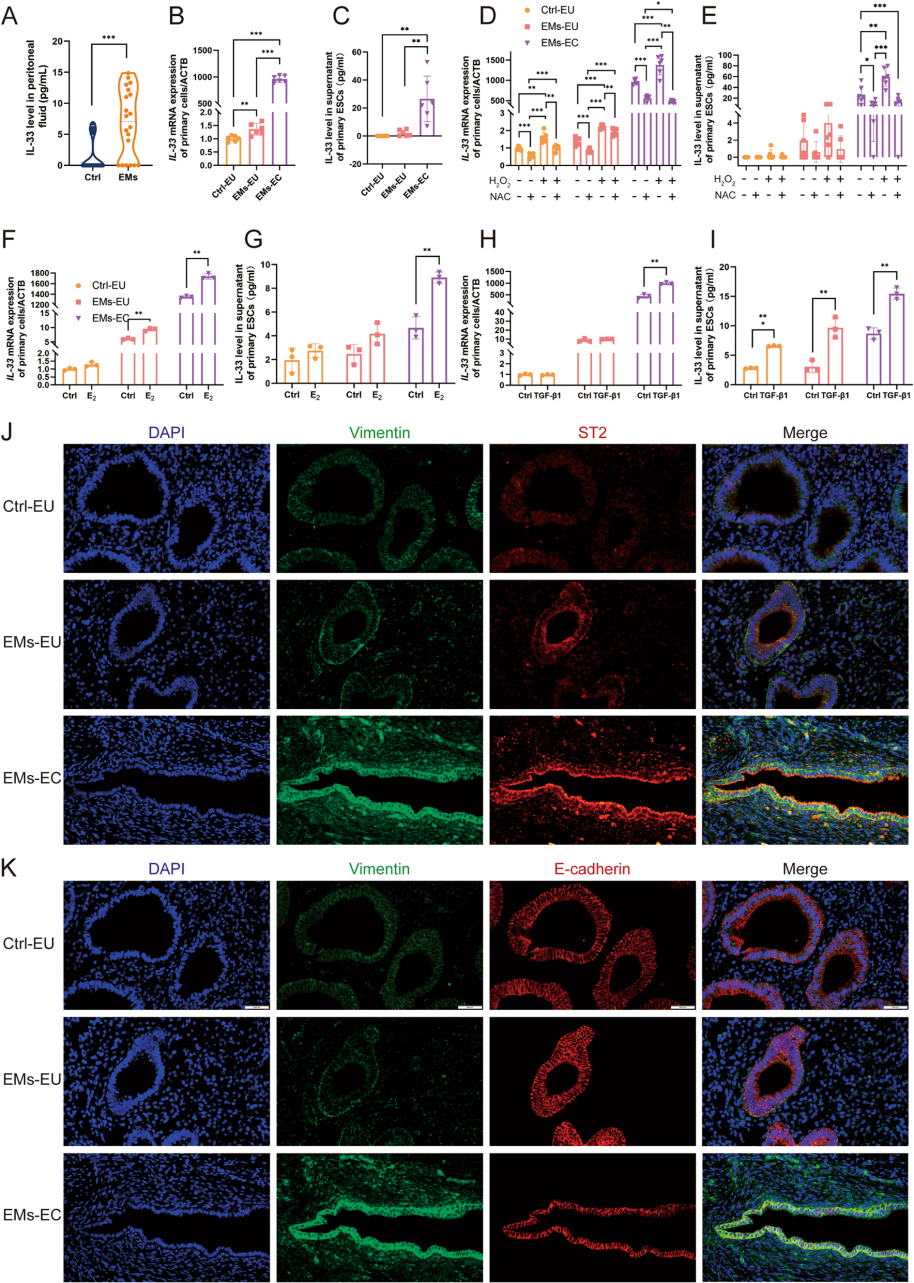
Expression Patterns and Inducing Factors of Interleukin-33 (IL-33) in Endometriotic milieu. **A** Levels of IL-33 were detected by ELISA in the peritoneal fluid of patients with EMs and controls. **B** Real-time PCR for analyzing the mRNA expression of IL-33 in primary endometrial stromal cells (ESCs). **C** Levels of IL-33 secretion in the cell supernatant of different primary ESCs. **D**-**I**: Changes of IL-33 expression in ESC after different treatments: mRNA (**D**) and protein (**E**) level of IL-33 after H_2_O_2_ and/or NAC treatment; mRNA (**F**) and protein (G) levels of IL-33 after estradiol (E_2_) treatment; mRNA (**H**) and protein (**I**) levels of IL-33 after TGF-β1 treatment. **J** Immunofluorescence co-localization of vimentin and ST2 (IL-33 receptor) in endometrial tissues. (blue:DAPI, green:vimentin, red: ST2) (Scale bars, 20 μm). **K** Immunofluorescence co-localization of vimentin and E-cadherin in endometrial tissues. (blue:DAPI, green:vimentin, red: E-cadherin) (Scale bars, 20 μm). Ctrl-EU, eutopic endometrium of controls; EMs-EU, eutopic endometrium of patients with endometriosis; EMs-EC, ectopic lesions. All data were analyzed using one-way *ANOVA* followed by *Dunnett’s post-hoc test*. Spearman’s correlation analysis was used to analyze the correlation between ST2 and vimentin expression in humans.* *P* < 0.05, ** *P* < 0.01, *** *P* < 0.001, data are presented as mean ± SEM

The circulating levels of IL-33 are elevated in patients with EMs than that in healthy individuals [29]. These findings indicate the vital role of IL-33 in the progression of EMs. To verify the expression of IL-33 in the endometriotic milieu, we performed ELISA on the peritoneal fluid of patients with or without EMs. These results confirmed the elevated IL-33 levels in the peritoneal cavity of patients with EMs (Fig. 1A).

To further investigate the source of IL-33 secretion in ectopic milieu, we cultured primary ESCs from the normal endometrium of controls and the eutopic endometrium and ectopic lesions of EMs patients simultaneously. The mRNA expression and protein secretion of IL-33 were then detected using real-time PCR and ELISA. Notably, IL-33 expression was significantly higher in ectopic ESCs than that in normal or eutopic ESCs (Fig. 1B, C).

Oxidative stress triggers IL-33 secretion during airway inflammation [30]. Similarly, ectopic endometriotic cells also survive in oxidative stress, hypoxic environments, transforming growth factor-β1 (TGF-β1) [31], and estradiol (E_2_) [32] have been recognized as essential factors in EMs development. Therefore, we explored whether these factors could trigger IL-33 secretion by ESCs. First, we confirmed that ectopic lesions and eutopic endometrium from EMs patients exhibited higher expression of 4-HNE, a classic oxidative product, indicating a high oxidative stress environment (Supplementary Fig. 1A), consistent with previous reports [33]. Next, we designed a treatment concentration gradient of H_2_O_2_ and determined the optimal non-lethal concentration of 1 μM based on cell viability assessed by CCK-8 and the level of ROS detected by the DCFH-DA probe (Supplementary Fig. 1B, C). Following H_2_O_2_ treatment for 24 h, primary ectopic ESCs exhibited increased ROS levels and MDA expression, along with decreased levels of peroxidase enzymes (superoxide dismutase, total glutathione peroxidase, and catalase) due to consumption (Supplementary Fig. 1D-F). Although H_2_O_2_ promoted IL-33 mRNA expression in both eutopic and ectopic ESCs, IL-33 secretion was induced exclusively in ectopic ESCs. Additionally, this effect was reversed by administration of the ROS scavenger N-acetylcysteine (NAC) (Fig. 1D, E, Supplementary Fig. 1G). Similarly, both TGF-β1 and E2 in the ectopic milieu significantly triggered the secretion of IL-33 in ectopic ESC (F ig. 1F-I).

### IL-33/ST2 in ectopic milieu facilitate incomplete EMT processes

Endometriotic lesions exhibit marked fibrotic characteristics, a well-documented hallmark of this condition [34]. Masson’s trichrome staining confirmed the extensive presence of collagen fibers within the ectopic lesions (Supplementary Fig. 2A). Furthermore, vimentin, a recognized marker of EMT, displayed widespread expression in ectopic lesions with a significantly higher intensity than that of the endometrium in the control group (Supplementary Fig. 2C). Surprisingly, ST2, the IL-33 receptor, exhibited an expression pattern similar to that of vimentin (Supplementary Fig. 2B). Correlation analysis revealed a linear positive relationship between ST2 and vimentin (Y = 0.9617 *X + 0.09133, R^2^ = 0.8094, *P* < 0.0001) (Supplementary Fig. 2E), indicating its potential role in fibrotic processes via EMT. Curiously, there is no difference of expression on E-cadherinn between the endometrium from control group and ectopic lesions from EMs group (Supplementary Fig. 2D). Immunohistochemical staining and immunofluorescence co-localization revealed that ST2 was broadly expressed in the endometrial epithelium and stroma (Fig. 1J-K, Supplementary Fig. 2B). Obvious ST2 expression was observed in ectopic lesion from EMs, compared with that in endometrium from non-EM controls (Supplementary Fig. 2B).

To elucidate the role of IL-33 secreted from ectopic milieu on endometrial cells, human recombinant IL-33 protein (rIL-33) was added to the culture medium of hESCs, EECs and 12Z at different concentrations (0, 1, 10, 50, 100 ng/ml). Then, we evaluated cell viability after 12, 24, 48 h to determine the optimal concentration and time parameters. Treatment with 10 ng/ml IL-33 for 24 h showed the most substantial increase in cell viability, and this concentration and time model was used in subsequent experiments (Supplementary Fig. 1H).

Furthermore, to explore the autocrine and paracrine effects of IL-33 on ESCs and endometrial epithelial cells (EECs), we investigated the effect of IL-33 on profibrotic or EMT-related marker expression in our subsequent studies. Notably, α-smooth muscle actin (α-SMA) expression was higher in ectopic ESCs than that of eutopic ESCs and normal ESCs, and it could be further induced by IL-33 stimulation (Supplementary Fig. 3A-C). The promoting effect of IL-33 closely resembled that of the classical pro-fibrotic factor TGF-β1. Furthermore, IL-33 enhanced the proliferation and migration of ESCs, particularly ectopic ESCs (Supplementary Fig. 3D-F).

**Fig. 2:**
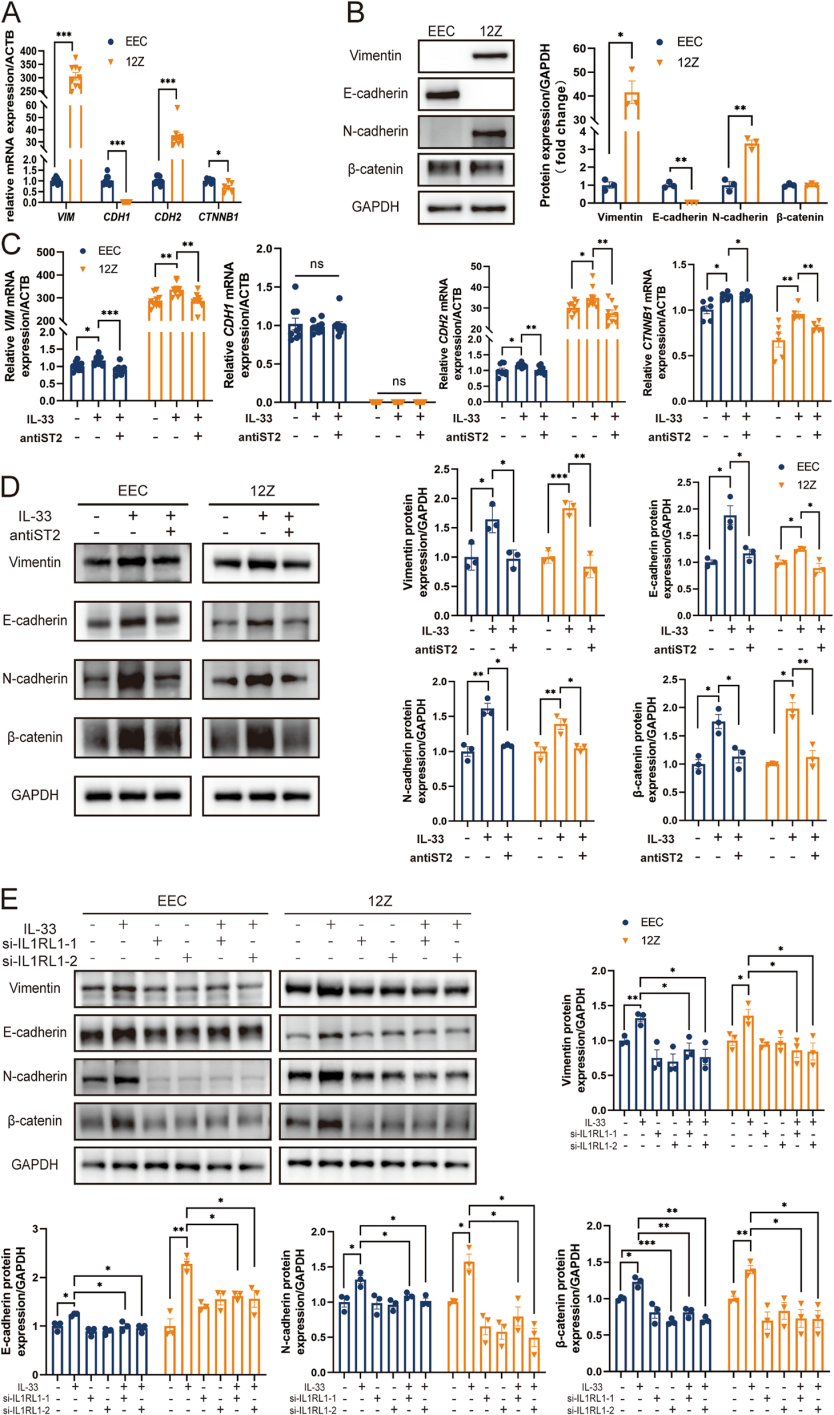
IL-33 is Crucial for the Epithelial–Mesenchymal Transition (EMT) Process in EECs. **A** RNA expression of EMT-related genes (*VIM, CDH1, CDH2,* and *CTNNB1*) in EECs and 12Z cells. **B** EMT-related protein (vimentin, E-cadherin, N-cadherin, and β-catenin) were detected using western blot in EECs and 12Z cells. C mRNA expression of EMT-related genes (*VIM, CDH1, CDH2,* and *CTNNB1*) in EECs and 12Z cells after IL-33 or/and ST2 neutralizing antibody treatment. **D** Protein expression of EMT-related genes (vimentin, E-cadherin, N-cadherin, and β-catenin) in EEC and 12Z treated by IL-33 or/and ST2 neutralizing antibody. **E** Protein expression of EMT-related genes (vimentin, E-cadherin, N-cadherin, and β-catenin) in EECs and 12Z cells treated by IL-33 or/and knocking down ST2 by siRNA. Data are presented as mean ± SEM. All data were analyzed using one-way *ANOVA* followed by *Dunnett’s *post hoc* test and Student’s t-test*; * *P* < 0.05, ** *P* < 0.01, *** *P* < 0.001

In comparison with normal and eutopic endometria, epithelial cells within ectopic lesions displayed elevated vimentin expression via IHC staining, indicative of obvious EMT characteristics (Fig. 1K, Supplementary Fig. 2C). Notably, the ectopic epithelial cell line 12Z exhibited more mesenchymal features than those of the normal endometrial epithelial cell line EEC. Specifically, 12Z displayed increased expression of stromal cell markers, such as vimentin and N-cadherin, along with reduced expression of the epithelial marker, E-cadherin (Fig. 2A, B). Interestingly, β-catenin, a classical transcription factor implicated in EMT, exhibited decreased RNA expression in 12Z, unchanged in protein (Fig. 2A, B). To investigate whether IL-33 promotes EMT in epithelial cells, we subjected both EEC and 12Z to IL-33 treatment for 24 h. IL-33 enhanced the expression of vimentin, N-cadherin, and β-catenin at both mRNA and protein levels. Importantly, this effect was reversed by the use of neutralizing antibodies targeting ST2 (Fig. 2C, D). Furthermore, the expression of the epithelial marker E-cadherin did not exhibit significant changes at the RNA level following IL-33 treatment; however, a slight increase in the protein level was observed. This intriguing finding suggests that IL-33 may induce an incomplete EMT process in EMs, which warrants further exploration. In addition to neutralizing antibodies, we achieved similar inhibitory results by knocking down ST2 using siRNA (Fig. 2E, Supplementary Fig. 4A).

### IL-33 derived from ectopic milieu increased CCN4 and Fra-1 expression

**Fig. 3:**
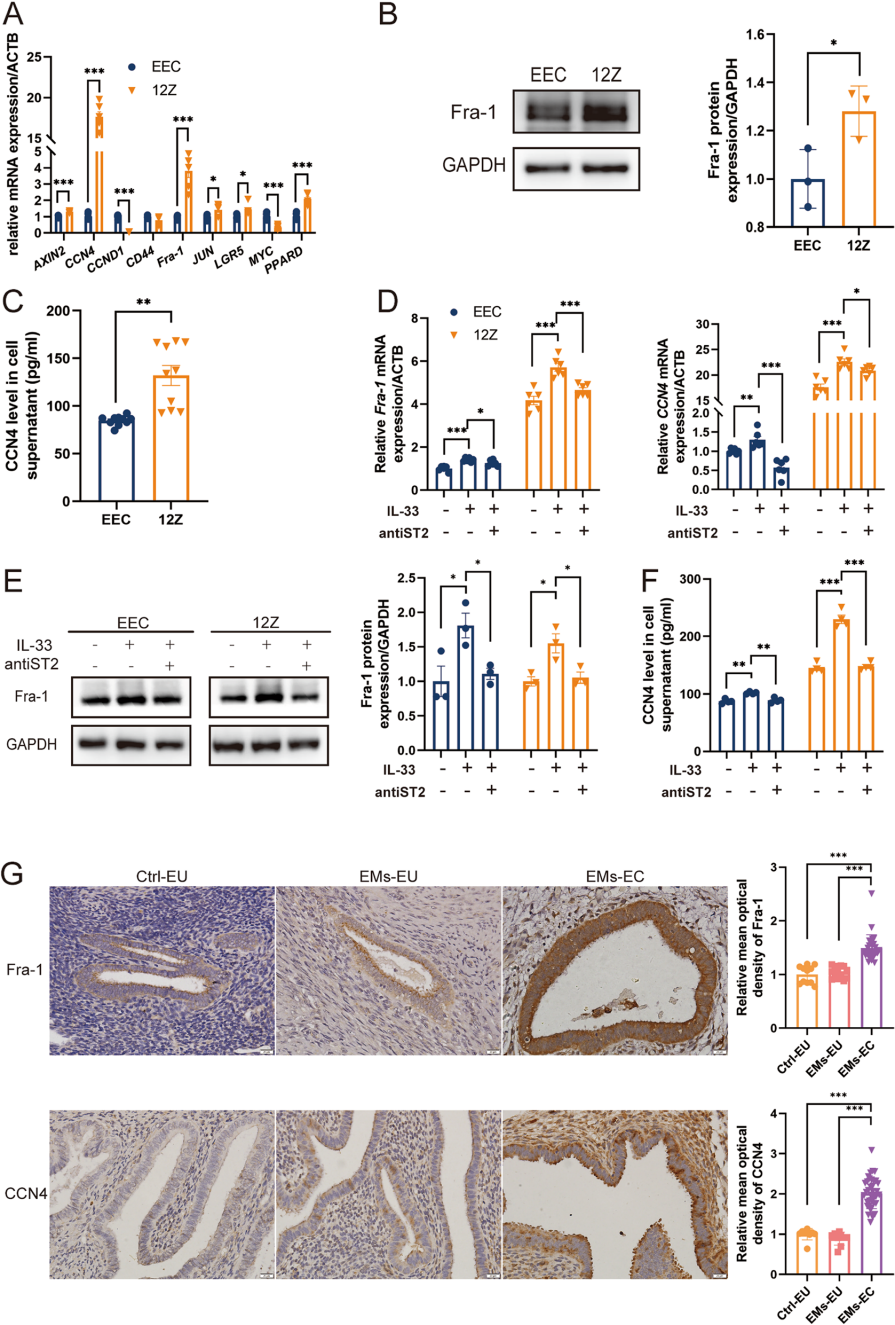
IL-33 Enhances the Expression of Downstream Target Genes of WNT/β-catenin Pathway. **A** RNA expression of downstream target genes of WNT/β-catenin (*AXIN2, CCN4, CCND1, CD44, Fra-1, JUN, LGR5, MYC, and PPARD*) were detected via real-time PCR in EECs and 12Z cells. **B** Fra-1 expression in EECs and 12Z cells was detected by western blotting. **C** CCN4 secretion in the cell supernatant of EECs and 12Z cells was detected via ELISA. **D**-**F** After IL-33 or/and ST2 neutralizing antibody treatment, the mRNA expression and protein levels of Fra-1 and CCN4 in EECs and 12Z cells. **G** Immunohistochemical staining of Fra-1 and CCN4 in eutopic endometrium and ectopic lesions. Data are presented as mean ± SEM. All data were analyzed using one-way *ANOVA* followed by *Dunnett’s *post hoc* test and Student’s t-test*; *P < 0.05, **P < 0.01, ***P < 0.001

Several pathways, including the RAS/RAF/MEK/ERK, PI3K/AKT/mTOR, and Wnt/beta-catenin pathways, play a role in promoting EMT. Among them, β-catenin is one of the most indispensable molecules for EMT. In this study, CCN4 and Fra-1, the downstream signal molecules of β-catenin, were screened for high expression in 12Z via real-time PCR (Fig. 3A). Correspondingly, the protein levels of CCN4 and Fra-1 (Fig. 3B-C) in 12Z were notably higher than in EEC. The results in the cell line were consistent with the results of IHC staining in TMA tissues (Fig. 3G). Fra-1 was reported to induce EMT in epithelial cells through direct binding of the TGFB1 and ZEB2 promoters as well as the first intron of ZEB1 and thereby regulating their expression [35]. Our correlation analysis revealed a linear positive relationship between CCN4 and collagen deposition (Y = 74.90 *X - 53.62, *R*^2^ = 0.8088, *P* < 0.0001) (Supplementary Fig. 2F). In addition, IL-33 exposure further enhanced the expression of CCN4 and Fra-1, and this effect was eliminated using neutralizing antibodies targeting ST2 (Fig. 3D-F).

### IL-33 phosphorylated β-catenin by PKA pathway, promoting CCN4 and Fra-1 expression

**Fig. 4:**
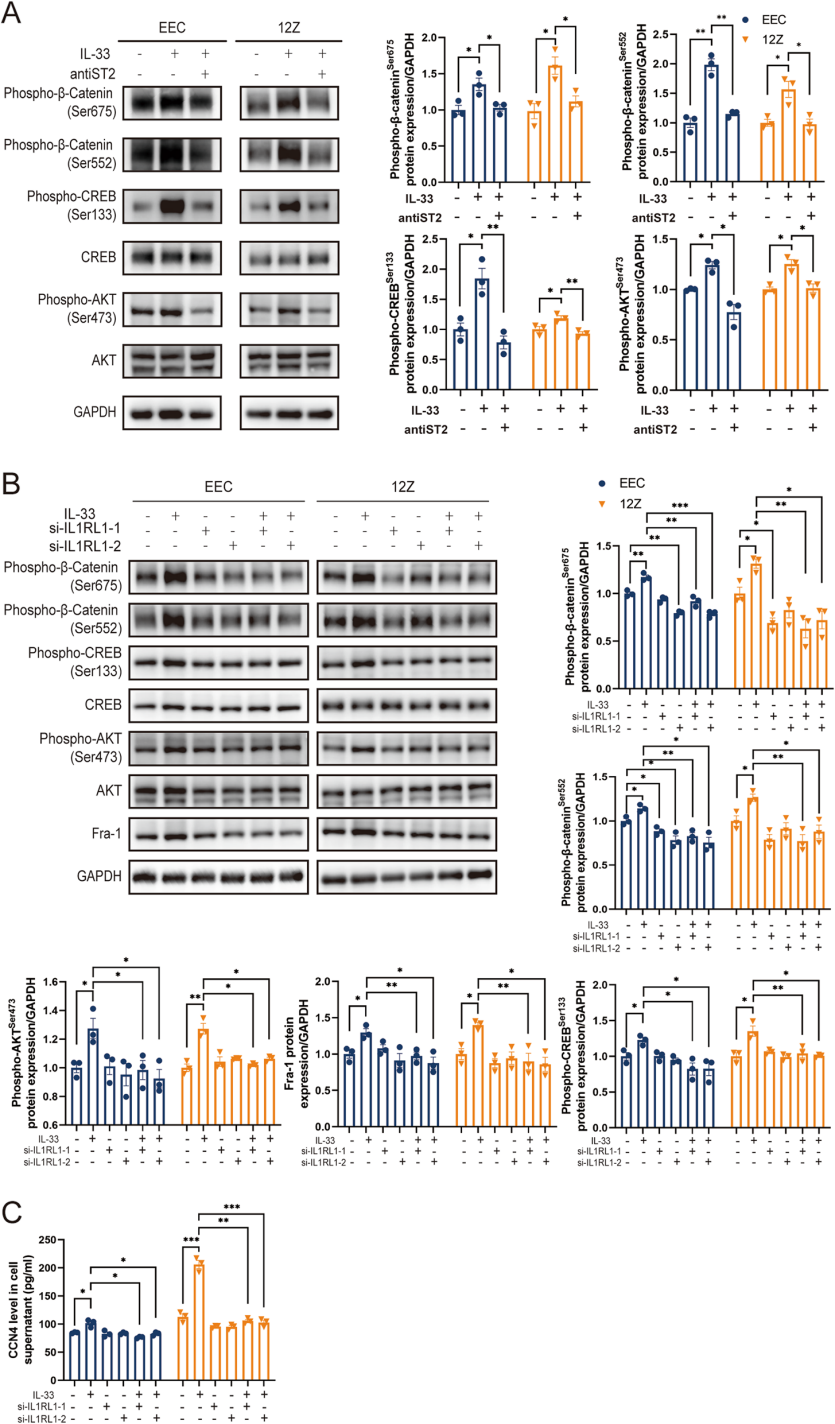
IL-33 obviously Induced β-catenin Phosphorylation. **A** Protein expression of phospho-β-catenin (Ser675 and Ser552), phospho-CREB (Ser133), CREB, phospho-AKT(Ser473) and AKT in EECs and 12Z cells treated by IL-33 or/and ST2 neutralizing antibody. **B** Protein expression of phospho-β-catenin (Ser675 and Ser552), phospho-CREB (Ser133), CREB, phospho-AKT (Ser473), AKT, and Fra-1 in EECs and 12Z cells treated by IL-33 or/and knocking down ST2 by siRNA. **C** Expression of CCN4 in supernatant of EEC and 12Z treated by IL-33 or/and knocking down ST2 by siRNA. Data are presented as mean ± SEM. All data were analyzed using one-way *ANOVA* followed by *Dunnett’s *post hoc* test and Student’s t-test*; * *p* < 0.05, ** *p* < 0.01, *** *p* < 0.001

We hypothesized that IL-33 could regulate β-catenin expression in a post-translationally modified manner. Reviewing the literature [36], we noticed that protein kinase A (PKA) and AKT (also known as protein kinase B) can enhance protein stability and inhibit degradation by phosphorylating the Ser675 and Ser552 sites of β-catenin. To confirm our hypothesis, we conducted assays within cells following IL-33 exposure. After 24 h of IL-33 stimulation, the expression of phospho-β-catenin (Ser675 and Ser552) in both EEC and 12Z strongly increased, along with phospho-AKT (Ser473) and phospho-CREB (Ser133), which served as an indirect marker for PKA activation (Fig. 4A, B). The effects of IL-33 on β-catenin were blocked by neutralizing antibodies against ST2 or ST2 knockdown via siRNA (Fig. 4).

**Fig. 5:**
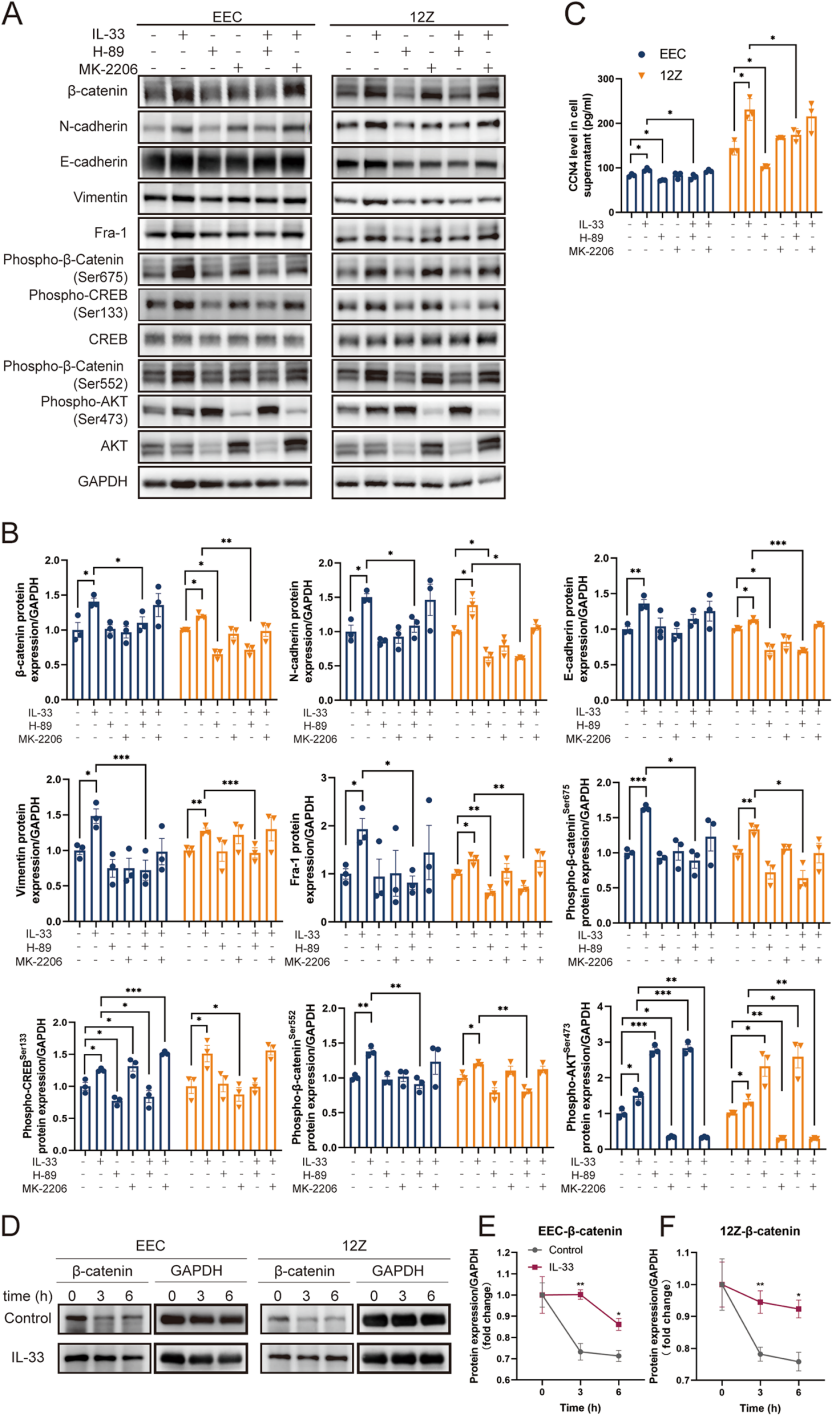
IL-33 Mediates β-catenin Phosphorylation at Ser675 and Ser552 by PKA. **A**, **B** Protein expression of EMT-related proteins (vimentin, E-cadherin, N-cadherin, and β-catenin), Fra-1,phospho-β-catenin (Ser675 and Ser552), phospho-CREB (Ser133), CREB, phospho-AKT (Ser473) and AKT in EEC and 12Z treated by IL-33, PKA inhibitor-H89 or/and AKT inhibitor- MK2206. **C** Expression of CCN4 in supernatant of EEC and 12Z treated by IL-33, PKA inhibitor- H89 or/and AKT inhibitor- MK2206. **D**, **F** Cyclohexane chase assay of β-catenin in EECs and 12Z cells treated by IL-33. Data are presented as mean ± SEM. All data were analyzed using one-way *ANOVA* followed by *Dunnett’s *post hoc* test and Student’s t-test*; * *P* < 0.05, ** *P* < 0.01, *** *P* < 0.001

To further confirm the role of IL-33 in promoting β-catenin phosphorylation through PKA and AKT, we inhibited PKA with H-89 and AKT with MK-2206. The results showed that H-89 not only inhibited IL-33-mediated CREB phosphorylation but also hindered β-catenin phosphorylation at both sites, leading to decrease in total β-catenin protein levels. Conversely, although MK-2206 inhibited AKT phosphorylation, it did not significantly impede β-catenin phosphorylation or decrease total β-catenin protein levels, especially in 12Z cells (Fig. 5A, B). Hence, we propose that IL-33-mediated β-catenin phosphorylation is primarily achieved via PKA rather than AKT, a slight departure from previously published findings [36]. Interestingly, H-89 significantly increased AKT phosphorylation at Ser473 while inhibiting PKA. Nevertheless, β-catenin phosphorylation was reduced, affirming that IL-33 modulates β-catenin primarily through PKA. As anticipated, H-89 also inhibited the expression of Fra-1 and CCN4, which are downstream target proteins of β-catenin and EMT-related factors (Fig. 5A-C). The CHX chase assay further confirmed the inhibition of β-catenin degradation by IL-33 (Fig. 5D-F).

### IL-33 Stabilized β-catenin to facilitate its function as a transcription factor, promoting the expression of CCN4 and Fra-1

**Fig. 6:**
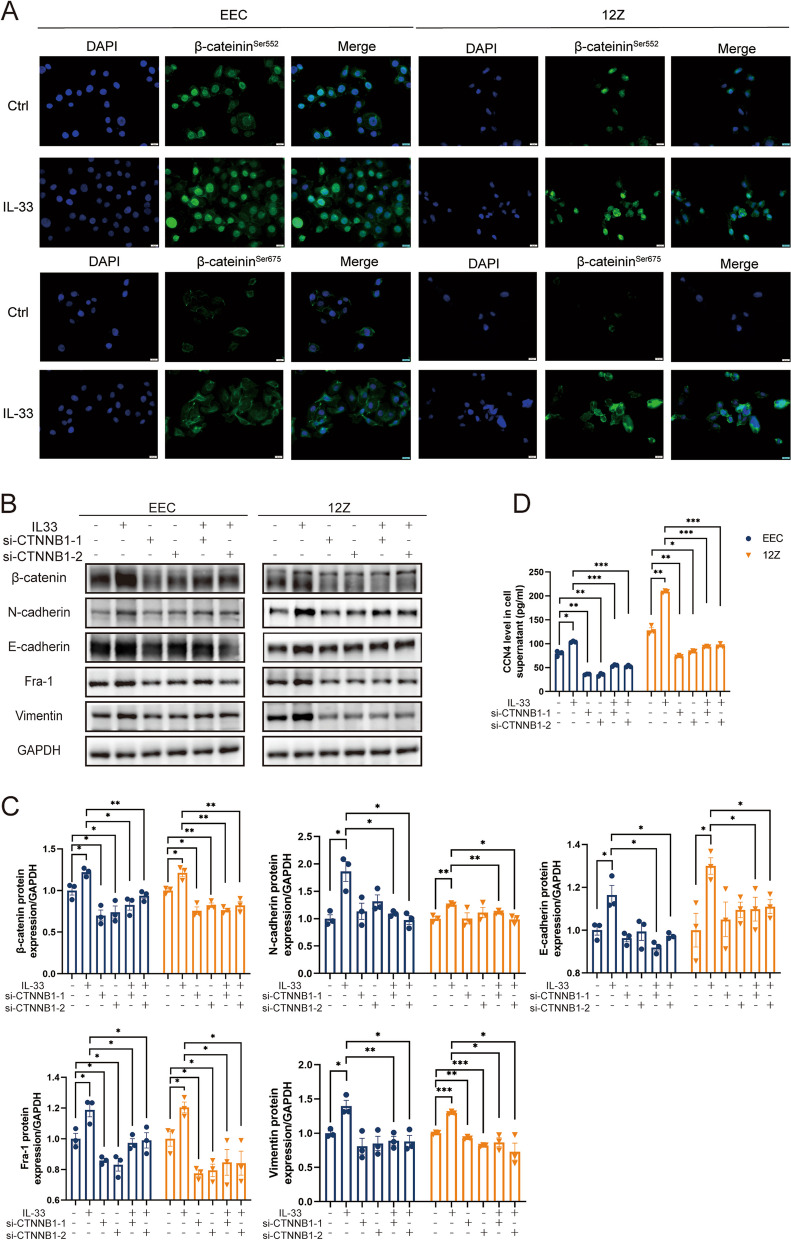
IL-33-ST2 Signal Blocking or β-catenin Knockout Weakens EMT in EEC and 12Z. **A**. Immunofluorescence staining of phospho-β-catenin at Ser675 and Ser552 (Scale bars, 20 μm). **B**, C Protein expression of EMT-related proteins (Vimentin, E-cadherin, N-cadherin, and β-catenin) and Fra-1 in EECs and 12Z cells treated by IL-33 or/and knocking β-catenin by siRNA. **D** Expression of CCN4 in supernatant of EEC and 12Z treated by IL-33 or/and knocking β-catenin by siRNA. Data are presented as mean ± SEM. All data were analyzed using one-way *ANOVA* followed by *Dunnett’s *post hoc* test and Student’s t-test*; * *P* < 0.05, ** *P* < 0.01, *** *P* < 0.001

In addition to changes in protein expression, we observed that β-catenin^Ser552^ was primarily located in the nucleus, whereas β-catenin^Ser675^ was predominantly cytoplasmic (Fig. 6A). These results establish that IL-33 promotes the stability of β-catenin protein, facilitating its function as a transcription factor in the nucleus and thereby promoting the expression of downstream target genes, such as Fra-1 and CCN4.

To further affirm β-catenin as a key downstream gene in IL-33-mediated EMT, we employed siRNA to knock down β-catenin(Supplementary Fig. 4B). The results demonstrated that the promotion of EMT-related protein expression by IL-33 was inhibited after β-catenin knockdown (Fig. 6B-D). These findings strongly suggest that IL-33 mediates EMT through the β-catenin signaling pathway.

### Profibrotic effect of IL-33 was verified in vivo

**Fig. 7:**
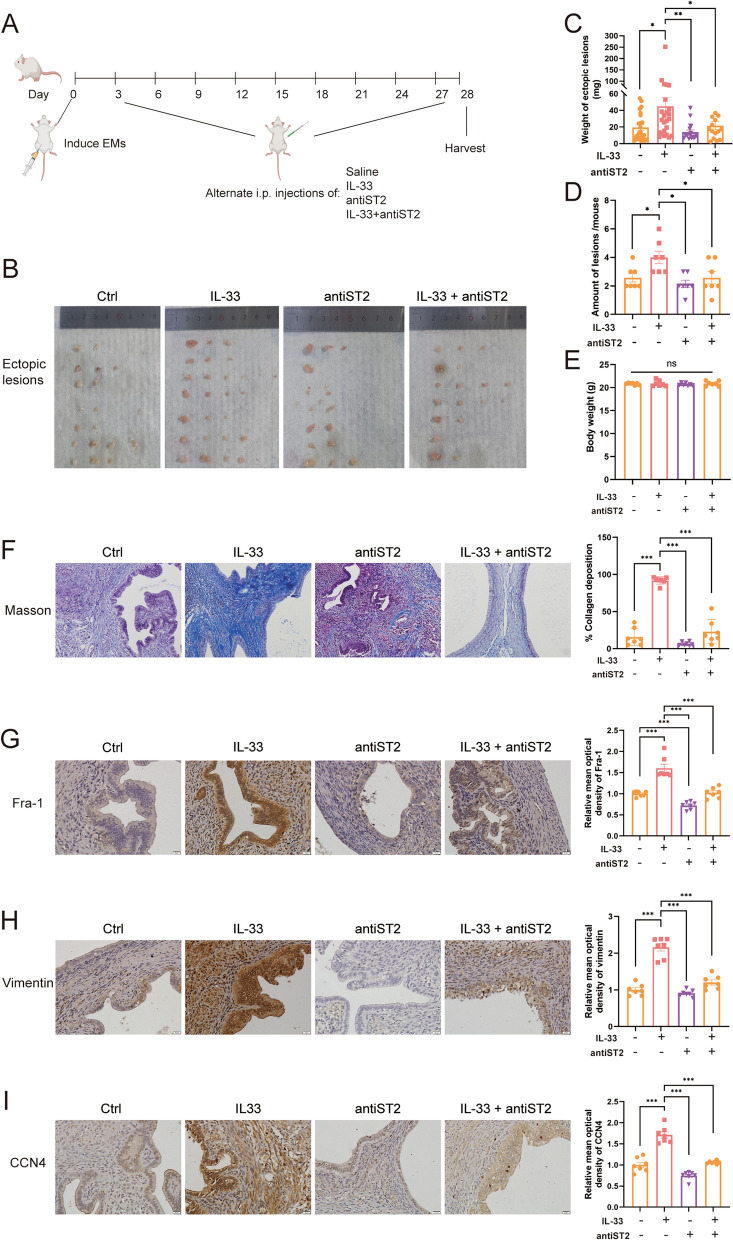
IL-33 Promotes Fibrosis via EMT in Allograft Mouse Model of Endometriosis. **A** Schematic representation of the experimental outline shows the induction of endometriosis (day 0), i.p. injections of saline, IL-33, antiST2 or IL33 + anti ST2 every two days (beginning on day 3), and euthanasia (day 28). **B** Macroscopic view of ectopic endometriotic lesions in each group of mouse model. **C**, **D** Weight and number of lesions in mice treated with IL-33 or/and antiST2. **E** Body weights of mice in different groups. **F**-**I**. Masson, Fra1, vimentin, and CCN4 staining of lesions in different groups of mice. Data are presented as mean ± SEM. All data were analyzed using one-way *ANOVA* followed by *Dunnett’s *post hoc* test and Student’s t-test*; * *p* < 0.05, ** *p* < 0.01, *** *p* < 0.001

**Fig. 8:**
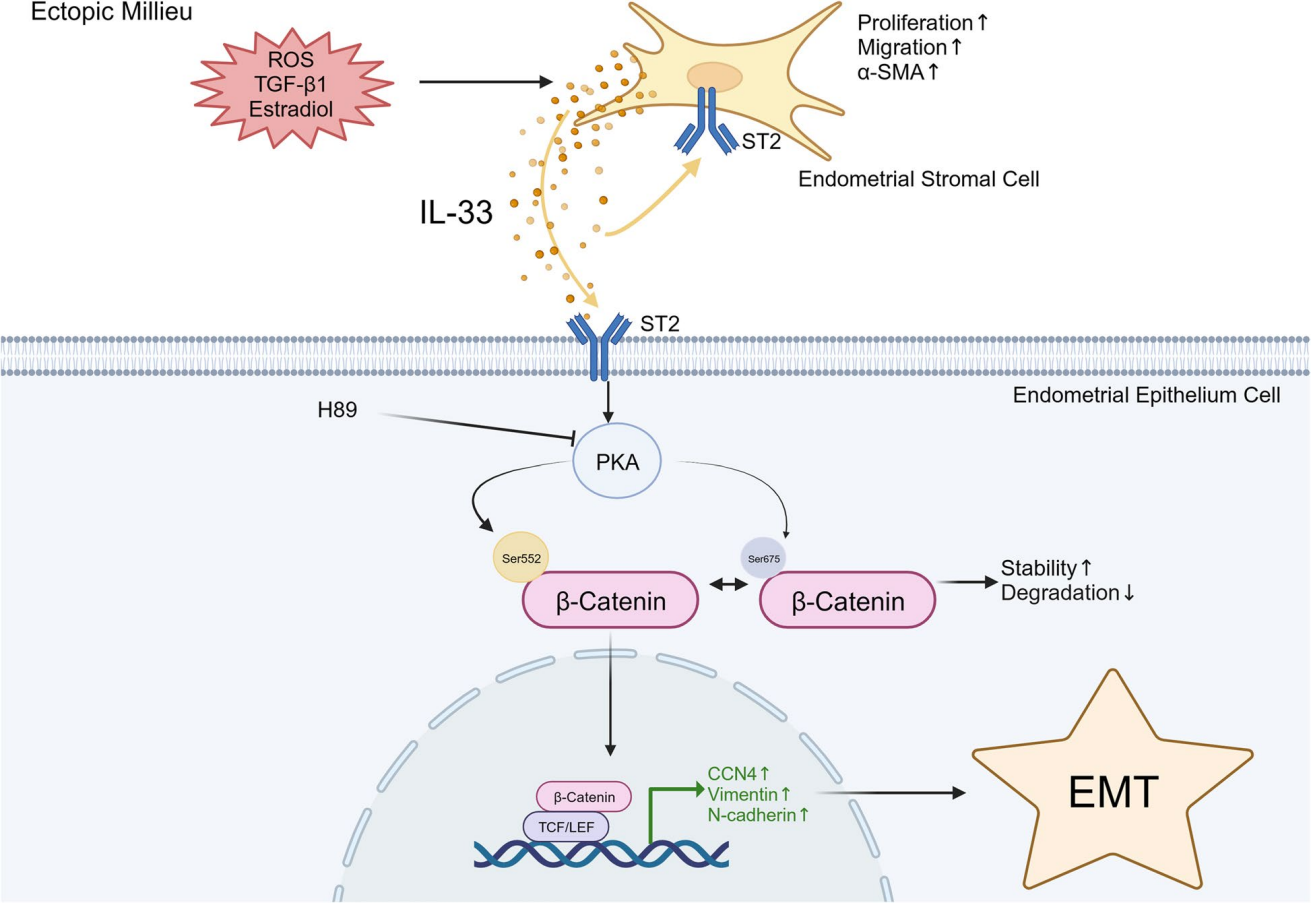
Schematic illustration of IL-33 Promoting EMT Process in Endometriotic Milieu. Interleukin 33 (IL-33) is highly expressed in ectopic ESCs, acting via the receptor ST2. Ectopic milieu, characterized by ROS, TGF-β1, and high level of estrogen, triggers secretion of IL-33 in ESCs, which in turn, enhanced the aggressive implantation and survival of ESCs. Meanwhile, elevated IL-33 in ectopic milieu also activated WNT/β-catenin pathway in EECs by phosphorylating β-catenin (Ser675 and Ser552), which primes exresssion of ECM related genes (CCN4 and Fra-1) and mesenchymal markers, enhanceing the EMT process and extracellular matrix produnction. Thus, IL-33/ST2 axis plays a pivotal role in endometriosis progress by promoting EMT. This figure was created with biorender.com

The mechanism underlying the effect of IL-33 on EMs is closely associated with EMT. Considering that EECs and ESCs constitutively express IL-33R, IL-33 is a potent activator of autocrine and paracrine signaling in endometrial cells, and IL-33 plays a critical regulatory role in EMs in vitro, we hypothesized that IL-33 could promote the EMT of EECs and thereby regulate the development of EMs-associated fibrosis. To validate this hypothesis, we performed in vivo experiments using an allograft mouse model of EMs. After implanting the endometrial fragments into the abdominal cavity, the mice were intraperitoneally injected with IL-33 and ST2-neutralizing antibodies every two days and harvested at 4 weeks (Fig. 7A). IL-33 application enhanced the success rate of modeling as well as the lesion number and weight (Fig. 7B-D). Using IHC, we found that IL-33 aggravated the degree of fibrosis and increased expression of EMT-related molecules and the downstream signal molecules of β-catenin in ectopic endometrial lesions, in which ECM components were significantly elevated (Figs. 7F-I and 8). Correlation analysis revealed a linear positive relationship between CCN4 and collagen deposition (Y = 125.6*X - 21.80, *R*^2^ = 0.8704, *P* < 0.0001) (Supplementary Fig. 2G). These effects are blocked by neutralizing antibodies targeting ST2. The in vivo results further established that IL-33 may promote EMT-related fibrosis through β-catenin-mediated expression of downstream molecules.

In summary, our results reveal that the cytokine IL-33, secreted by ESCs, plays a crucial role in triggering EMT by phosphorylating β-catenin in EECs. This accumulation of IL-33 facilitates better survival of ectopic ESCs in the context of oxidative stress or inflammation, thereby facilitating the progression of EMs and related fibrosis. Moreover, we used allogeneic transplantation to establish a mouse model of EMs and employed treatment with IL-33 antibodies or ST2 inhibitors.

## Discussion

The progression of EMs is associated with the development of inflammation and fibrosis. Fibrosis is a prominent characteristic of EMs, leading to the formation of scars and nodules that affect the physiological functions of the affected organs [2]. First-line clinical hormone therapy can provide temporary pain relief; however, clear amelioration of fibrosis and adhesions, the main causes of disease manifestation and recurrence, by hormone therapy has not yet been reported. Ayako et al. recently reported that a long-acting antibody against the cytokine IL-8 not only reduced the size of endometriotic lesions but also ameliorated fibrosis and adhesions [37]. Therefore, targeting specific fibrosis is of interest in treatment research and may play an important role in influencing treatment outcomes [38]. EMT is a crucial component of the fibrotic process observed in EMs [39]. We observed noticeable collagen deposition and elevated expression of vimentin in ectopic lesions than that observed in the eutopic endometrium. Ectopic epithelial 12Z cells expressed higher levels of mesenchymal markers than EECs, indicating a distinct EMT state. Consistent with previous research, our data indicated that the EMT transition may not occur before retrograde menstruation but rather after the formation of EMs [40], possibly influenced by the local microenvironment. Therefore, identifying the key factors inducing epithelial cells to undergo EMT may be crucial for relieving EMT-related fibrosis.

Furthermore, ROS [3], estrogen [41] and TGF-β1[42]derived from the ectopic endometrial milieu induced the secretion of IL-33 by ESCs. IL-33, in turn, enhanced the proliferation, migration, and expression of α-SMA in ESC, shaping an autocrine feedback loop. IL-33 serves as a key mediator in the regulation of inflammation in various diseases, including its role in regulating group 2 innate lymphoid cells implicated in cardiac fibrosis [15, 19]. Similarly, upregulation of the IL-33/ST2 axis may contribute to tubular cell injury and fibrosis via EMT in the kidneys [14, 43]. Lin et al*.* found that IL-33 upregulates MMP-9 expression in ESCs via the ST2/MAPK signaling pathway, enhancing their invasive capability [21]. Furthermore, the volume of endometriotic lesion was significantly reduced in Il33^−/−^ and Il1r1^−/−^ mice and almost completely suppressed in Myd88^−/−^ mice [44]. Although emerging data support a novel contribution of IL-33/ST2 signaling to the remodeling and differentiation processes of fibrosis, there is still room for better understanding.

Because IL-33 could be used as an early marker for ulcer-associated activated fibroblasts and myofibroblast trans-differentiation [14], it’s the potential role in EMs-associated fibrosis cannot be ruled out. Similarly, upregulation of the IL-33/ST2 axis may contribute to fibrosis via EMT of ectopic EECs in the context of periodic bleeding and damage repair. Consistent with our prediction, IHC staining of the IL-33 receptor ST2 revealed diffuse expression of ST2 in both the eutopic endometrium and ectopic lesions, with particularly high levels in the epithelium. After IL-33 treatment, a significant elevation in mesenchymal markers was observed in both EEC and 12Z cells, suggesting its contribution to EMT. Furthermore, IL-33 synchronously upregulated the expression of mesenchymal and epithelial markers, indicating a partial EMT but not a complete state. This observation implied that fibrosis in EMs is not a terminal outcome but rather an ongoing and actively progressive pathological process [2].

Notably, β-catenin levels substantially increased upon IL-33 exposure, especially in ectopic epithelial 12Z cells. The Wnt/β-catenin pathway is a classic pathway involved in EMT [39, 45]. Fra-1 and CCN4, the downstream target genes of β-catenin, were also elevated. It is known that CCN4 plays an important role in embryonic development, wound healing, and tissue repair. Recent research find CCN4 under pathological state is correlated with expression of fibrosis markers, potentially facilitate to obesity-associated liver fibrosis [46], also contributes to lung [47] and kidney fibrosis [48]. Furthermore, Fra-1 participates in EMT in cancer cells by driving the expression of EMT-inducing transcription factors [49]. However, the exact mechanisms by which IL-33 affects Wnt/β-catenin signaling may vary depending on the cell type and context, and whether IL-33/ST2 signaling affects β-catenin stabilization or nuclear translocation in EMs remains unclear. In our study, we further confirmed that IL-33 phosphorylated β-catenin at Ser552 and Ser675 by activating PKA. The increased phosphorylation of β-catenin, along with its increased protein stability and induced translocation into the cell nucleus, enhanced the expression of downstream target genes (Fra-1 and CCN4), promoting EMT process and ECM components production. In EMs, IL-33 demonstrated disease specificity in the phosphorylation pattern of β-catenin and its subsequent nuclear translocation after phosphorylation. IL-33 commonly functions through the MyD88/TRAF6 and PI3K/AKT pathways [50, 51]. For the phosphorylation of β-catenin, Ser675 is phosphorylated by PKA, whereas Ser552 is phosphorylated by AKT [36]. However, our study revealed that IL-33 has an activating effect on both PKA and AKT. Specifically, in both EEC and 12Z, phosphorylation of β-catenin at both the 552 and 675 sites was facilitated by PKA. The application of signal AKT inhibitor MK2206 failed to suppress IL-33-induced β-catenin phosphorylation. Additionally, phosphorylated β-catenin at Ser675 primarily accumulated in the cytoplasm, whereas phosphorylation at Ser552 resulted in nuclear translocation, distinctive from other diseases [52]. The knockout of β-catenin in cell line provides additional evidence on the role of IL-33 in EMs-related fibrosis through the β-catenin pathway. Emerging evidence regarding IL-33 and Wnt/β-catenin crosstalk may hold the key to unraveling the complex pathogenesis of EMs.

Additionly, tissue-derived immune cells, such as ILC2s, mast cells and Tregs, express the ST2 receptor constitutively and are major targets of IL-33 in vivo [53, 54]. Furthermore, IL-33 also activates additional subsets, such as Th2 cells, basophils, eosinophils, and macrophages [11, 53, 55, 56]. Recent studies have shown that IL-33 directly affected differentiation of macrophage subset, which mediated the resolution of inflammation [57], tissue repair[58]or drug-resistant properties in cancer [59].In early pregnancy, decidual macrophages (dMφs) from recurrent spontaneous abortion (RSA) patients presented a M1 phenotype with high secretion of IL-33 and decreased expression of ST2. IL-33/ST2 axis modulates the polarization and efferocytosis of decidual macrophages (dMφs) [60], lead to pregnancy failure [61].Macrophage-derived IL-33 also upregulates SLC7A11 in ectopic endometrial stromal cells and protects against ferroptosis in eESC, accelerating the progression of endometriosis [62]. Thus, these findings highlight some potential etiological mechanism of IL-33 in pathological conditions whereby IL-33 axis might perturbs immune homeostasis by interfering with macrophage function in microenvironment.

In conclusion, our research offers in vitro and in vivo evidence to unravel the complexities and implications of IL-33 in EMs, shedding light on its potential interaction with the Wnt/β-catenin pathway. Targeting the IL-33/ST2 axis may offer promising strategies for managing EMs-associated symptoms, relieving fibrosis, and hindering disease progression.

Our study in a tertiary medical institutions (Center for Endometriosis) also has some limitations.Due to delayed clinical visits or patients referred from primary hospitals, most of the samples collected in this study are from patients with severe endometriosis. In addition, it is well known that polarized macrophage, activated neutrophils, and tissue-derived cytokine, as key regulators that link the fibrosis in a variety of diseases [63, 64]. *Macrophages and neutrophils are also the most infiltrated immune cells in ectopic endometrial tissue, and the cross-talk between IL-33 and macrophage was not involved in this study, which may be limited for our profound understanding of pathogenic mechanism mediated by IL-33. Future studies using IL-33(*^*−/−*^*) mice or IL-33 receptor-deficient (ST2*^*−/−*^*) mice may gain a more comprehensive understanding of IL-33 in EMT process during endometriosis progression and fibrosis*.

## Summary

In conclusion, our research offers in vitro and in vivo evidence to unravel the complexities and implications of IL-33 in endometriosis, shedding light on its potential interaction with the Wnt/β-catenin pathway. Targeting the IL-33/ST2 axis may be a promising strategy for managing endometriosis-associated symptoms, relieving fibrosis, and stopping disease progression.

## Statistical analysis

The continuous variables are presented as the mean ± SEM. Data from two groups were analyzed by Student’s t-test, whereas data from multiple groups were analyzed using one-way ANOVA using Tukey’s post-hoc test. Spearman’s correlation analysis was used to analyze the correlation between ST2 and vimentin expression in humans. Statistical analyses were performed using Statistical Package for the Social Sciences 26.0 software (SPSS Inc., Chicago, USA) and Prism 5.0 software (GraphPad Software Inc.). A *P*-value < 0.05 indicated statistical significance.

### Supplementary Information

Additional file 1.

Additional file 2.

Additional file 3.

Additional file 4.

Additional file 5: Supplementary Figure 1. Establishment of a Non-lethal Oxidative Stress Model in vitro. A Expression of 4-HNE is in endometrium and ectopic lesions (Scale bars, 20 μm)  B-C: Cell viability (B) and fluorescence density (C) of reactive oxygen species (ROS) in ESC treated with different concentration gradients of H2O2. D-F Typical fluorescence picture of ROS(D), expression of malondialdehyde (E), superoxide dismutase, total glutathione peroxidase and catalase (F) in ESC treated with 1μM H2O2. G Secretion of IL-33 at different times after treatment with 1 μM H2O2 in eutopic ESC and ectopic ESC. H Cell lines hESCs, EECs and 12Z were respectively treated with human recombinant IL-33 protein (rIL-33) in vitro at different concentrations (0, 1, 10, 50, 100 ng/ml) and time nodes. Then, the cell viability was determined for the optimal concentration and time parameters of rIL-33 via CCK8. Ctrl-EU, eutopic endometrium of controls; EMs-EU, eutopic endometrium of patients with endometriosis; EMs-EC, ectopic lesions. Data are presented as mean ± SEM. All data were analyzed using one-way ANOVA followed by Dunnett’s post hoc test and Student’s t-test; * *p* < 0.05, ** *p* < 0.01, *** *p* < 0.001.

Additional file 6: Supplementary Figure 2. Immunohistochemical staining of ST2, Vimentin, and E-cadherin. A Percentage of collagen area to total area in eutopic endometrium and ectopic lesion from controls and EMs patients as determined by Masson staining (Scale bars, 100 μm). B-D Relative mean optical densities of ST2 (IL-33 receptor), vimentin, and E-cadherin as determined by immunohistochemical (IHC) staining (Scale bars, 20 μm). E Simple linear regression of ST2 and vimentin expressions (Y = 0.9617 * X + 0.09133, *R*^2^ = 0.8094, *P* < 0.0001). F Simple linear regression of percentage of collagen deposition and CCN4 expressions in human samples (Y = 0.7490 *X - 53.62, *R*^2^ = 0.8088, *P* < 0.0001). G Simple linear regression of percentage of collagen deposition and CCN4 expressions in mouse sample (Y = 125.6 * X - 21.80, *R*^2^ = 0.8704, *P* < 0.0001).

Additional file 7: Supplementary Figure 3. Autocrine Effect of IL-33 on Stromal Cells. A-C. mRNA (A) and protein (B, C) expression of α-SMA in eutopic ESCs and ectopic ESCs treated with IL-33 or TGF-β1. D-F Cell viability (D) and migration (E, F) of eutopic ESC and ectopic ESC treated with IL-33. Ctrl-EU, eutopic endometrium of controls; EMs-EU, eutopic endometrium of patients with endometriosis; EMs-EC, ectopic lesions. Data are presented as mean ± SEM. All data were analyzed using one-way ANOVA followed by Dunnett’s post hoc test and Student’s t-test; * *p* < 0.05, ** *p* < 0.01, *** *p* < 0.001.

Additional file 8: Supplementary Figure 4. Knockdown of ST2 and β-catenin Induced by siRNA were Confirmed by WB. A Protein expression of ST2 in EECs and 12Z cells treated by knocking ST2 by siRNA. B Protein expression of β-catenin in EECs and 12Z cells treated by knocking β-catenin by siRNA.

Additional file 9.

## Data Availability

No datasets were generated or analysed during the current study.
