# Supplementary material for: The IL-33-ST2 axis plays a vital role in endometriosis via promoting epithelial–mesenchymal transition by phosphorylating β-catenin
Source: Cell Commun Signal. 2024 Jun 10;22:318. doi: 10.1186/s12964-024-01683-x (PMC11163813; doi:10.1186/s12964-024-01683-x)
Supplement: Supplementary file 4 — Additional file 4. [file 12964_2024_1683_MOESM4_ESM.docx]

**Supplementary Material**

**Supplementary Table 4**

**The statistics of clinical parameters in patients with or without EMs**

| **Mean±SEM** | **Non-EMs (n=22)** | **EMs (n=26)** | ***p*** |
| --- | --- | --- | --- |
| Age (y) | 37.95±1.25 | 35.12±1.56 | 0.172 |
| BMI (kg/m^2^) | 21.69±0.56 | 21.67±0.67 | 0.520 |
| Menstrual cycle | | | |
| Proliferative phase, n (%) | 13(59.09) | 14(53.85) | 0.715 |
| Secretory phase, n (%) | 9(40.91) | 12(46.15) |  |
| VAS scores | 0.23±0.16 | 4.96±0.63 | ＜0.001 |
| Lesion location | | | |
| Ovary, n (%) | / | 3(11.54) | / |
| DIE, n (%) | / | 0 |  |
| Ovary and DIE, n (%) | / | 23(88.46) |  |
| Stage of revised American Fertility Society (rAFS, 1985) | | | |
| I-II, n (%) | / | 2(7.69) | / |
| III-IV, n (%) | / | 24(92.31) |  |
| Adenomyosis | | | |
| Yes, n (%) | / | 24(92.31) | ＜0.001 |
| No, n (%) | 22(100%) | 2(7.69) |  |
| CA125 (U/mL) | 18.51±1.74 | 38.05±1.34 | ＜0.001 |
| AMH (ng/ml) | 2.16±0.31 | 2.66±0.50 | 0.397 |

*Continuous variables were described by mean ± SEM; Categorical variables were described by quantity and percentage.*
