# Supplementary figures and images for: The IL-33-ST2 axis plays a vital role in endometriosis via promoting epithelial–mesenchymal transition by phosphorylating β-catenin
Source: Cell Commun Signal. 2024 Jun 10;22:318. doi: 10.1186/s12964-024-01683-x (PMC11163813; doi:10.1186/s12964-024-01683-x)

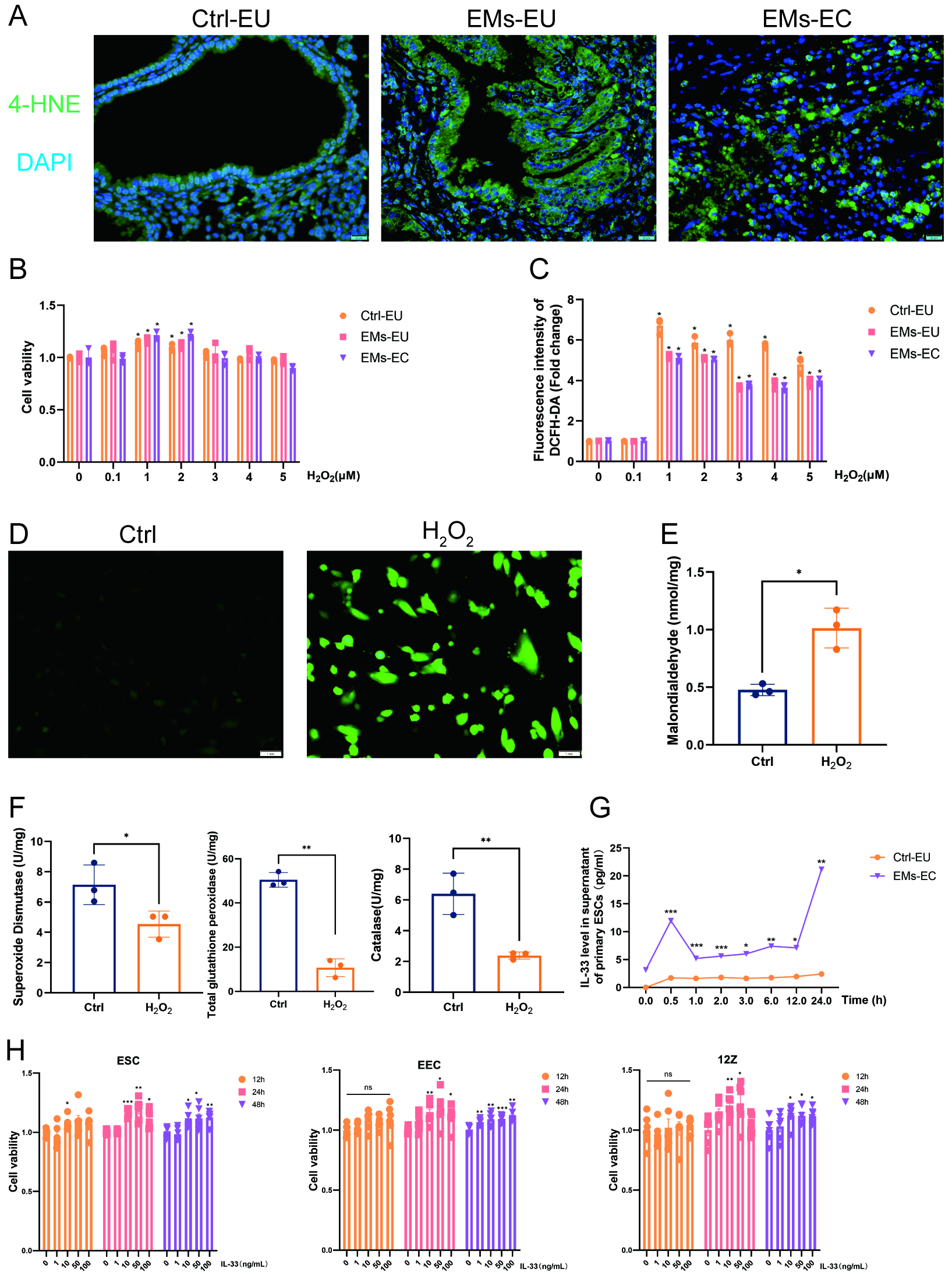

Supplement: Supplementary file 5 — Additional file 5: Supplementary Figure 1. Establishment of a Non-lethal Oxidative Stress Model in vitro. A Expression of 4-HNE is in endometrium and ectopic lesions (Scale bars, 20 μm) B-C: Cell viability (B) and fluorescence density (C) of reactive oxygen species (ROS) in ESC treated with different concentration gradients of H2O2. D-F Typical fluorescence picture of ROS(D), expression of malondialdehyde (E), superoxide dismutase, total glutathione peroxidase and catalase (F) in ESC treated with 1μM H2O2. G Secretion of IL-33 at different times after treatment with 1 μM H2O2 in eutopic ESC and ectopic ESC. H Cell lines hESCs, EECs and 12Z were respectively treated with human recombinant IL-33 protein (rIL-33) in vitro at different concentrations (0, 1, 10, 50, 100 ng/ml) and time nodes. Then, the cell viability was determined for the optimal concentration and time parameters of rIL-33 via CCK8. Ctrl-EU, eutopic endometrium of controls; EMs-EU, eutopic endometrium of patients with endometriosis; EMs-EC, ectopic lesions. Data are presented as mean ± SEM. All data were analyzed using one-way ANOVA followed by Dunnett’s post hoc test and Student’s t-test; * p < 0.05, ** p < 0.01, *** p < 0.001. [file 12964_2024_1683_MOESM5_ESM.tif]

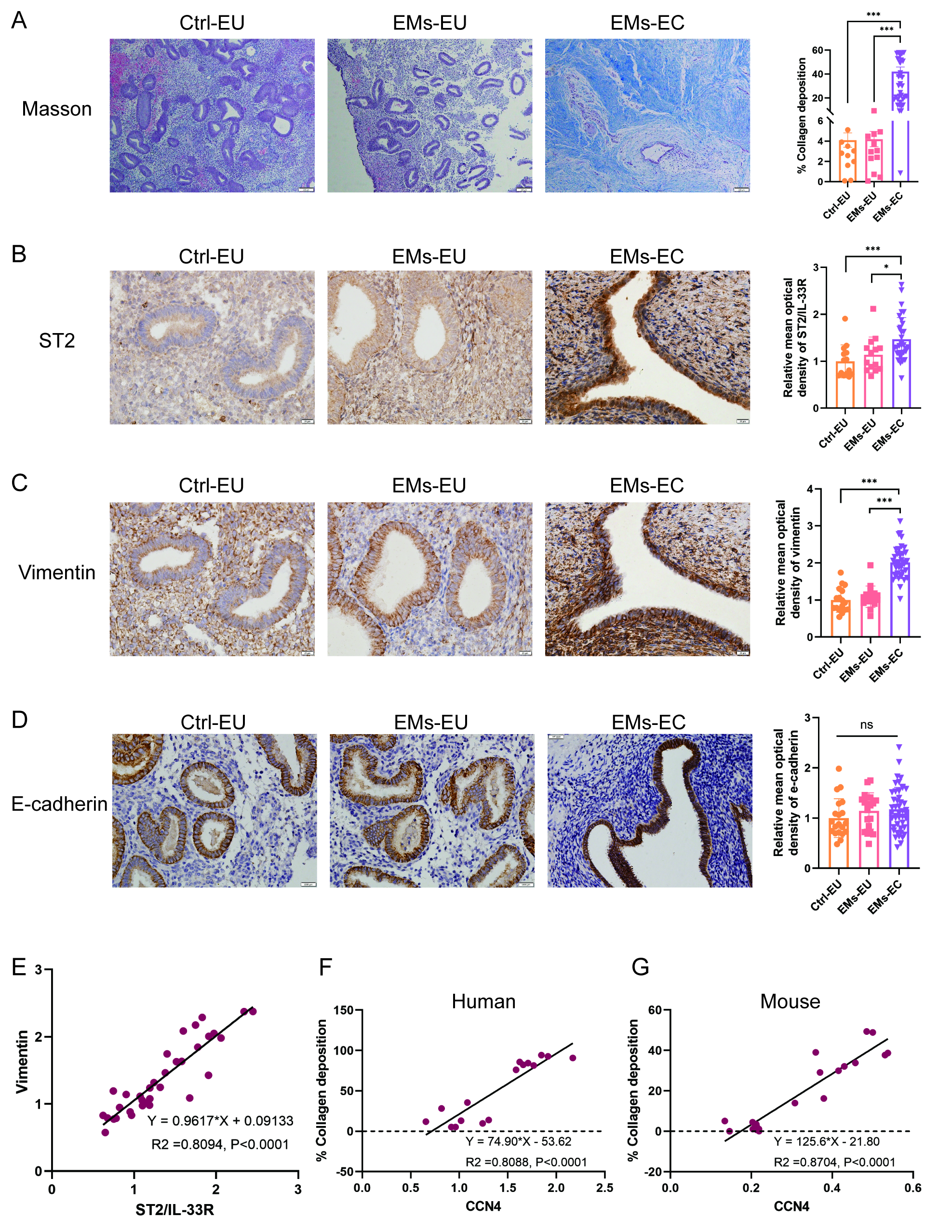

Supplement: Supplementary file 6 — Additional file 6: Supplementary Figure 2. Immunohistochemical staining of ST2, Vimentin, and E-cadherin. A Percentage of collagen area to total area in eutopic endometrium and ectopic lesion from controls and EMs patients as determined by Masson staining (Scale bars, 100 μm). B-D Relative mean optical densities of ST2 (IL-33 receptor), vimentin, and E-cadherin as determined by immunohistochemical (IHC) staining (Scale bars, 20 μm). E Simple linear regression of ST2 and vimentin expressions (Y = 0.9617 * X + 0.09133, R2 = 0.8094, P < 0.0001). F Simple linear regression of percentage of collagen deposition and CCN4 expressions in human samples (Y = 0.7490 *X - 53.62, R2 = 0.8088, P < 0.0001). G Simple linear regression of percentage of collagen deposition and CCN4 expressions in mouse sample (Y = 125.6 * X - 21.80, R2 = 0.8704, P < 0.0001). [file 12964_2024_1683_MOESM6_ESM.tif]

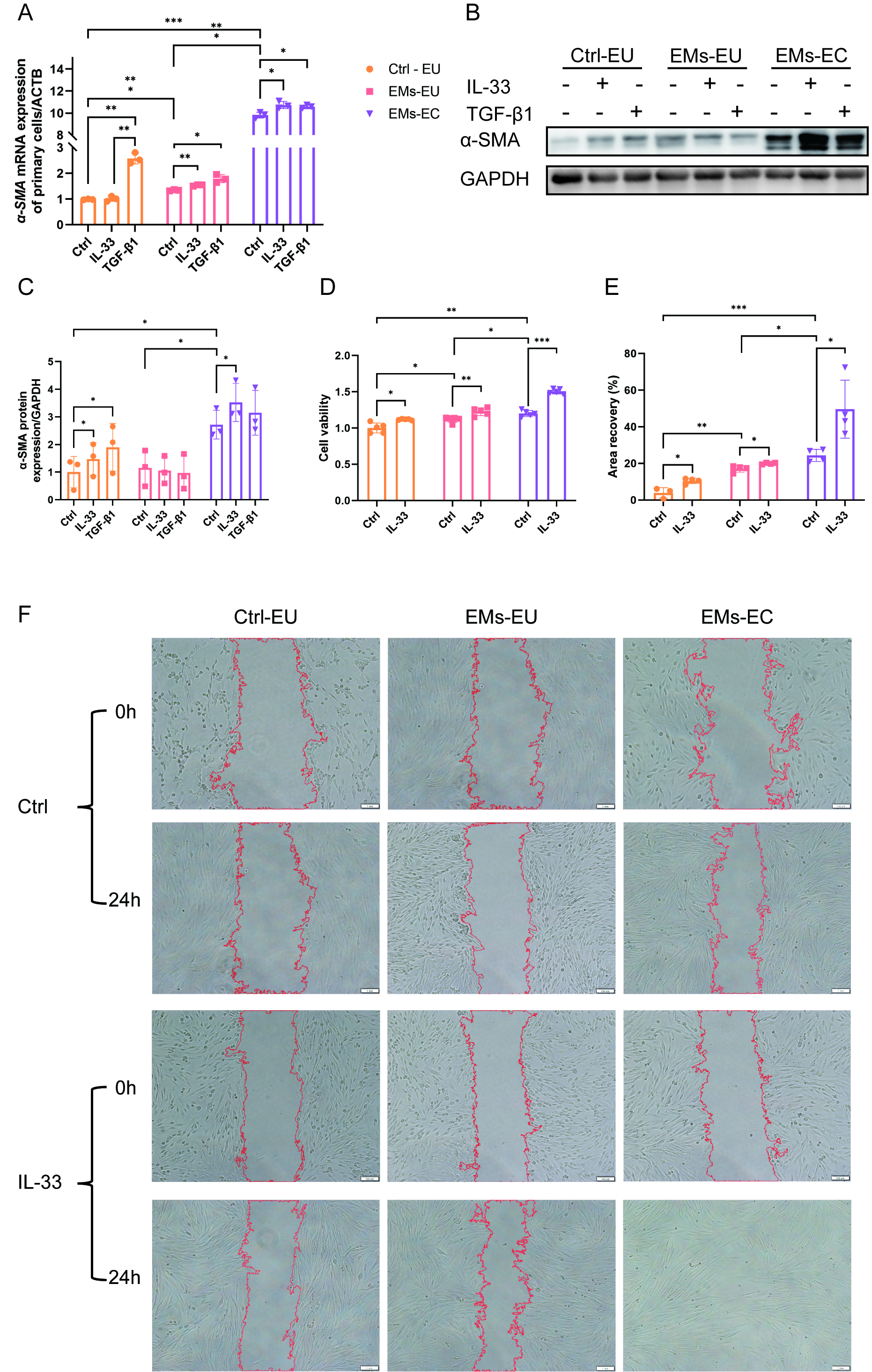

Supplement: Supplementary file 7 — Additional file 7: Supplementary Figure 3. Autocrine Effect of IL-33 on Stromal Cells. A-C. mRNA (A) and protein (B, C) expression of α-SMA in eutopic ESCs and ectopic ESCs treated with IL-33 or TGF-β1. D-F Cell viability (D) and migration (E, F) of eutopic ESC and ectopic ESC treated with IL-33. Ctrl-EU, eutopic endometrium of controls; EMs-EU, eutopic endometrium of patients with endometriosis; EMs-EC, ectopic lesions. Data are presented as mean ± SEM. All data were analyzed using one-way ANOVA followed by Dunnett’s post hoc test and Student’s t-test; * p < 0.05, ** p < 0.01, *** p < 0.001. [file 12964_2024_1683_MOESM7_ESM.tif]

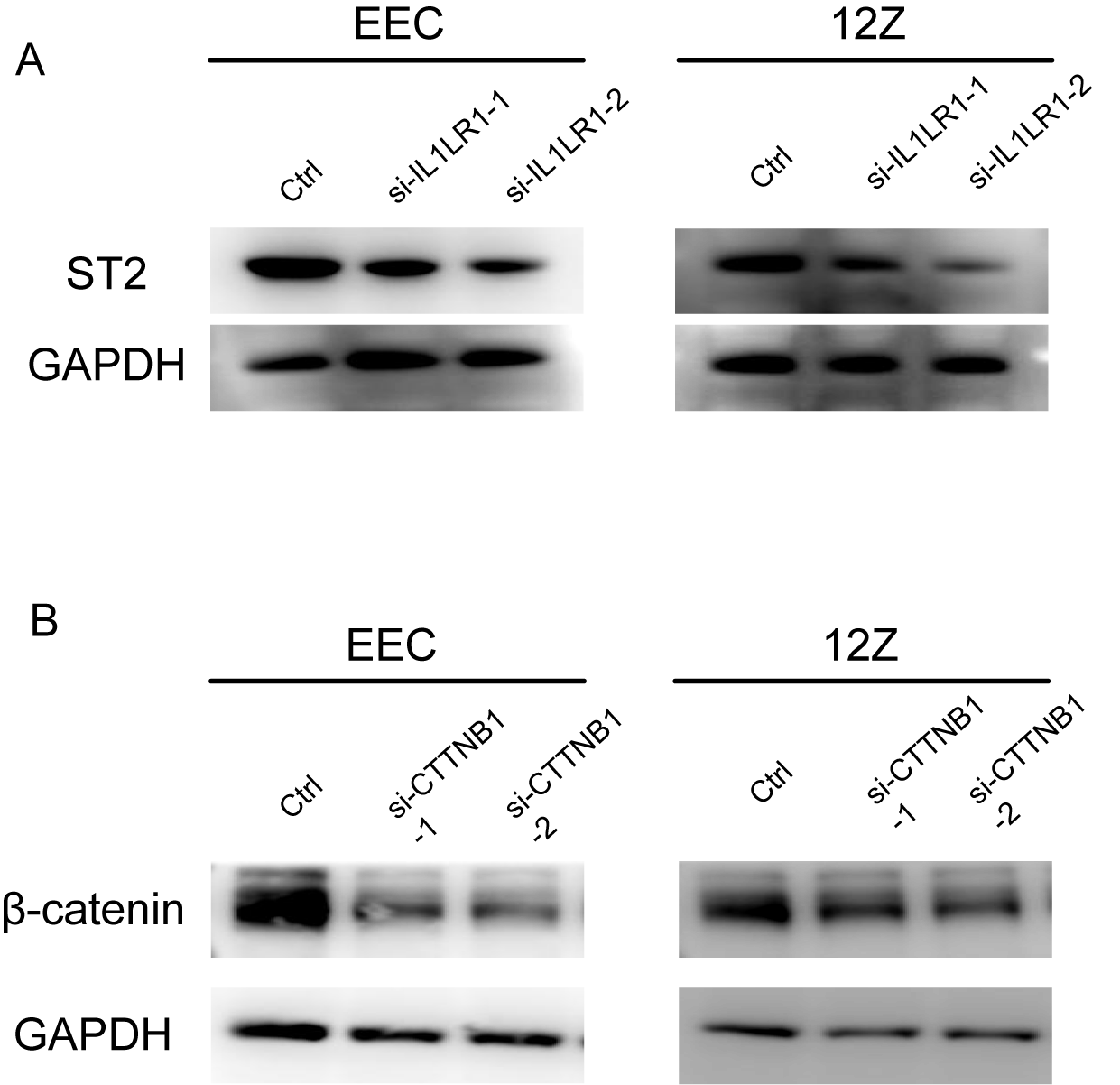

Supplement: Supplementary file 8 — Additional file 8: Supplementary Figure 4. Knockdown of ST2 and β-catenin Induced by siRNA were Confirmed by WB. A Protein expression of ST2 in EECs and 12Z cells treated by knocking ST2 by siRNA. B Protein expression of β-catenin in EECs and 12Z cells treated by knocking β-catenin by siRNA. [file 12964_2024_1683_MOESM8_ESM.tif]
